# Combined Transoral Robotic Tongue Base Surgery and Palate Surgery in Obstructive Sleep Apnea Syndrome: Modified Uvulopalatopharyngoplasty versus Barbed Reposition Pharyngoplasty

**DOI:** 10.3390/jcm10143169

**Published:** 2021-07-18

**Authors:** Yung-An Tsou, Chun-Chieh Hsu, Liang-Chun Shih, Tze-Chieh Lin, Chien-Jen Chiu, Vincent Hui-Chi Tien, Ming-Hsui Tsai, Wen-Dien Chang

**Affiliations:** 1 Department of Otolaryngology Head and Neck Surgery, China Medical University Hospital, Taichung 40402, Taiwan; d22052121@gmail.com (Y.-A.T.); jayhsu0522@gmail.com (C.-C.H.); entdrshih7111@gmail.com (L.-C.S.); drofarmyhospital@yahoo.com.tw (T.-C.L.); blueness1103@hotmail.com (C.-J.C.); vincenttien0623@asia.edu.tw (V.H.-C.T.); minghsui5121@gmail.com (M.-H.T.); 2 School of Medicine, China Medical University, Taichung 40402, Taiwan; 3 Department of Audiology and Speech-Language Pathology, Asia University, Taichung 41354, Taiwan; 4 Graduate Institute of Biomedical Sciences, China Medical University, Taichung 40402, Taiwan; 5 Department of Sport Performance, National Taiwan University of Sport, Taichung 404401, Taiwan

**Keywords:** obstructive sleep apnea, uvulopalatoplasty, barbed repositioning pharyngoplasty, transoral robotic surgery tongue base reduction

## Abstract

Background: Successful surgery outcomes are limited to moderate to severe obstructive sleep apnea (OSA) syndrome. Multilevel collapse at retropalatal and retroglossal areas is often found during the drug-induced sleep endoscopy (DISE). Therefore, multilevel surgery is considered for these patients. The aim of our study was to survey surgical outcomes by modified uvulopalatoplasty (UPPP) plus transoral robotic surgery tongue base reduction (TORSTBR) versus barbed repositioning pharyngoplasty (BRP) plus TORSTBR. Methods: The retrospective cohort study was performed at a tertiary referral center. We collected moderate to severe OSA patients who were not tolerant to positive pressure assistant PAP from September 2016 to September 2019; pre-operative–operative Muller tests all showed retropalatal and retroglossal collapse; pre-operative Friedman Tongue Position (FTP) > III, with the tonsils grade at grade II minimum, with simultaneous velum (V > 1) and tongue base (T > 1), collapsed by drug-induced sleep endoscopy (DISE) under the VOTE grading system. The UPPP plus TORSTBR (*n* = 31) and BRP plus TORSTBR (*n* = 31) techniques were offered. We compare the outcomes using an Epworth sleepiness scale (ESS) questionnaire, and measure the patients’ apnea–hypopnea index (AHI), lowest O_2_ saturation, cumulative time spent below 90% (CT90), and arousal index (AI) by polysomnography six months after surgery; we also measure their length of hospital stay and complications between these two groups. Results: Comparing BRP plus TORSTBR with UPPP plus TORSTBR, the surgical success rate is 67.74% and 38.71%, respectively. The significantly higher surgical success rate in the BRP plus TORSTBR group was noted. The surgical time is shorter in the BRP plus TORSTBR group. The complication rate is not significant in pain, bleeding, dysgeusia, dysphagia, globus sensation, and prolonged suture stay, even though the BRP plus TORSTBR rendered a higher percentage of globus sensation during swallowing and a more prevalent requirement of suture removal one month after surgery. The length of hospital stay is not significantly different between the two groups. Conclusion: In conclusion, BRP plus TORSTBR is a considerable therapy for moderate to severe OSA patients with DISE showing a multi-level collapse in velum and tongue base area. The BRP technique might offer a better anterior–posterior suspension vector for palate level obstruction.

## 1. Introduction

Patients with obstructive sleep apnea (OSA) often have breathing problems in their sleep due to partial or complete upper airway obstruction. In clinical research, the incidence of symptomatic OSA in male patients was higher than that in females, and the male to female prevalence ratio of OSA was 8:1 [1]. OSA often accompanied circulatory system diseases, such as coronary heart disease, hypertension, and heart failure [2]. There were also reported correlations of OSA to diabetes, Parkinsonism, Alzheimer’s disease, or dementia [3,4,5,6]. In fact, olfactory disorders were demonstrated as being associated with OSAS, with a significant linear correlation of threshold, discrimination, and identification (TDI) parameters and apnea–hypopnea index (AHI) [7]. Sleep surgery is important to improve life quality and decrease the symptoms of OSA patients [8]. Surgical treatment is one strategy to reduce the obstruction in OSA. Sleep surgery removes the obstructive tissue and enhances the cross-sectional airway area [8]. Surgeries for OSA focus on the management of the tongue base, which is an anatomic target, and remove the retroglossal airway and oropharyngeal obstructions in OSA patients [9]. Transoral robotic surgery (TORS) is a novel surgical technique for OSA patients and provides a visual assistant in targeted tissue operation for surgeons [10]. In addition, it also provides access to the retrolingual area, allowing the removal of the results of recurrent lingual tonsillitis, which, in patients who previously underwent tonsillectomies, can considerably reduce the air space [11].

Uvulopalatopharyngoplasty (UPPP) is a commonly performed surgery for OSA. Undergoing UPPP, the OSA patients had their tonsils resected and their uvula and soft palate removed [12]. A previous study presented that UPPP with transoral robotic tongue base reduction (TORSTBR) had the same rate of success as other surgical techniques, i.e., coblation tongue base resection and upper airway stimulation, and had clinical effects on the improvements in AHI, lowest O_2_ saturation, and the Epworth sleepiness scale (ESS) for OSA [13]. Lan et al. recommended that TORSTBR combined with UPPP could effectively reduce disease severity in patients with moderate to severe OSA [14]. However, UPPP demonstrated important fibrotic and stenotic complications secondary to the method; therefore, the procedure should be considered carefully for OSAS treatment. The tongue base is currently a crucial factor for moderate and severe OSA and could be effectively treated by TORSTBR. TORSTBR combined palatal surgery is also widely accepted by sleep surgeons all around the world, which provides better surgical results [15,16]. The barbed repositioning pharyngoplasty (BRP) is a recent surgical technique, and using a barbed suture allows for uninterruption of the muscular and mucosal structures [17]. BRP is a quick surgical procedure and is considered safe, feasible, and effective for OSA [17]. However, the surgical outcome is limited in moderate to severe OSA patients, since most of them have multilevel obstructions, including simultaneous retropalatal and retrolingual obstructions [18]. The appropriate surgical treatment should be multilevel, and there is still a lack of suitable surgical techniques for UPPP or BRP with TORSTBR in moderate to severe OSA patients. Therefore, we performed two kinds of multilevel surgery for moderate to severe OSA patients, including transoral robotic tongue base reduction with simultaneous different palatal surgeries by BRP or UPPP plus TORSTBR, and we compared the functional outcome and success rate in the patients with moderate to severe OSA.

## 2. Methods

### 2.1. Study Procedures

We conducted a retrospective case series with two comparative groups (UPPP plus TORSTRB, BRP plus TORSTBR) to survey these two surgical outcomes for patients with moderate to severe sleep apnea syndrome. This study was registered with the Research Ethics Committee of China Medical University and Hospital. Clinically, all of the patients were enrolled because of sleep apnea with loud snoring and symptoms of daytime sleepiness. Patients included in the study were 20 years or older, had an AHI over 15, and had more than three months postoperative polysomnography to diagnose their OSA as moderate to severe. All the PSG was performed overnight in a CMUH sleep center (Level I sleep study). Physical examinations revealed at least grade II enlarged tonsils, Grade III Mallampati score, and a thick soft palate with an elongated uvula [19]. Drug-induced sleep endoscopy was performed for all the patients. All patients revealed vellum anterior–posterior collapse over 50% and oropharyngeal lateral 50% collapse, reaching the Friedman grade II of lingual tonsils hypertrophy without epiglottic collapse by drug-induced sleep endoscopy (DISE) under the VOTE grading system [20]. In addition, all patients underwent PSG, which revealed at least moderate and severe OSA. The patients without a bulky tongue or those who were diagnosed to have mild sleep apnea were excluded from our study. Following diagnosis, all patients underwent multilevel surgery for managing multilevel obstruction by TORSTBR surgery with simultaneous palatal surgery by barbed suspension pharyngoplasty or modified UPPP in CMUH from September 2016 to September 2019. All patients who underwent either UPPP plus TORSTBR or BRP plus TORSTBR were enrolled. Informed consent for surgery was signed by both the sleep surgeon and patients.

### 2.2. Participants

**Figure 1:**
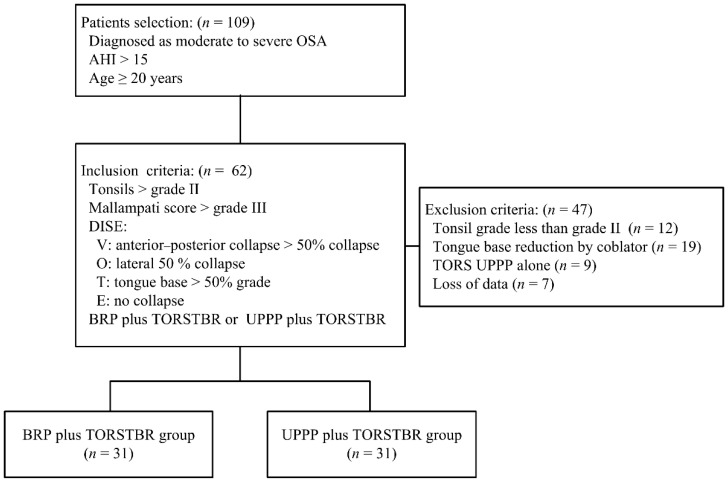
The flow chart of the current study. OSA, obstructive sleep apnea; AHI, apnea–hypopnea index; BRP, barbed repositioning pharyngoplasty; TORSTBR, transoral robotic tongue base reduction; UPPP, uvulopalatopharyngoplasty.

The 109 charts of OSA patients undergoing BRP or UPPP plus TORSTBR from September 2016 to September 2019 were reviewed. The included participants (*n* = 62) were informed of the study process, and informed consent for a retrospective review of their medical records was obtained before the study. We retrospectively reviewed patients who underwent BRP plus TORSTBR (*n* = 31) and UPPP plus TORSTBR (*n* = 31) groups. All of the tongue base volume resected was at least over 3 mL in both groups. (Figure 1). All participants and one researcher, a statistician, who analyzed the outcomes data, were unaware of the two surgical methods in this study.

### 2.3. Surgical Technique of BRP and UPPP

**Figure 2:**
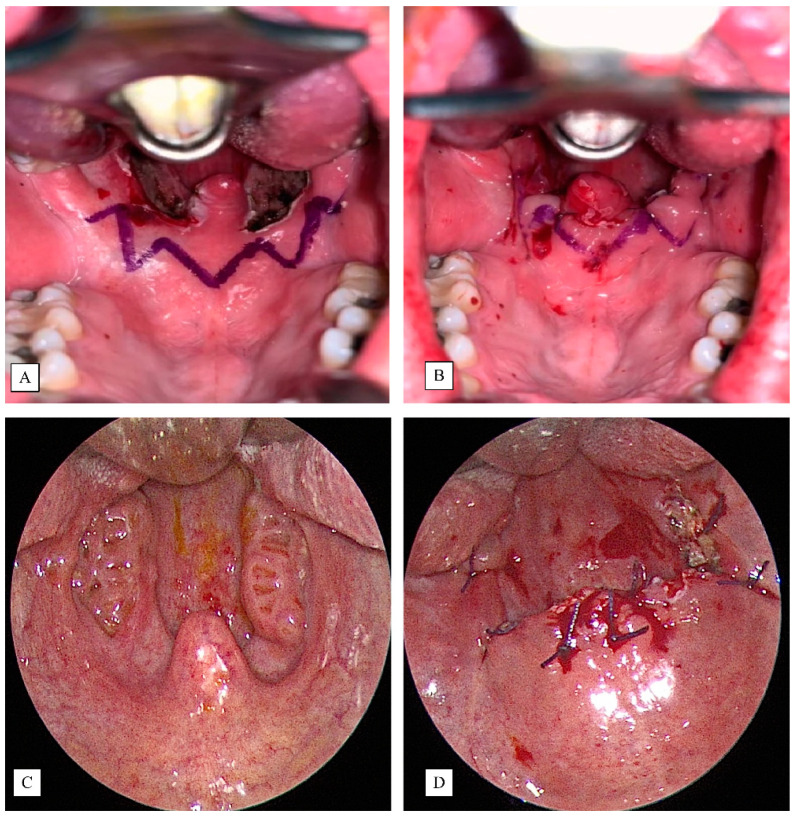
Before (**A**) and after (**B**) barbed suspension pharyngoplasty in the BRP plus TORSTBR group; before (**C**) and after (**D**) modified UPPP in the UPPP plus TORSTBR group.

In the BRP plus TORSTBR group (Figure 2A,B), barbed suspension pharyngoplasty was performed using a barbed suture V-Loc™ wound closure device in the soft palate for increasing anterior–posterior and lateral space velum and stiffness of the soft palate. We used two V-Loc sutures and started bidirectional suturing after tonsillectomy from the posterior nasal spine (midline of the junction of the soft palate and hard palate) through to the posterior pillar and back to the soft palate, reintroducing the needle close to the point of exit toward to pterygomandibular raphe near maxillary tuberosity, and then the lateral pharyngeal wall, and repeatedly anchoring to the pterygomandibular raphe [17]. The procedure was repeated on the other side. The palatopharyngeal muscle was neither divided nor repositioned.

In the UPPP plus TORSTBR group (Figure 2C,D), the surgery was under general anesthesia, and the patient was put in a supine position with a shoulder roll for neck extension. The Crowe–Davis mouth gag was applied for mouth opening. After a good surgical view is gained, the same was secured for the bilateral. Tonsillectomy was performed first by incising a 1 cm anterior tonsillar pillar cut above the upper pole of the palatine tonsil using a #15 blade and then dissecting the tonsillar capsule off the underlying palatal pharyngeal muscles [21]. A cold knife instrument was used, and the bleeder was stopped by bipolar electrocautery. Then, we preserved the posterior tonsillar pillar for less tension by suturing the posterior tonsillar pillar to the anterior tonsillar pillar by 3-0 vicryls sutures interruptedly from the upper tonsillar fossa towards the tongue. The uvulectomy was not routinely performed, except in instances where there was a longer uvula that was redundant to the tongue base. Most of the uvulas were not resected and preserved in our UPPP group patients.

### 2.4. TORSTBR

All patients underwent TORSTBR under general anesthesia by nasotracheal intubation [22,23]. The tongue base was exposed by a laryngeal advanced retractor system (Fentex, Tuttlingen, Germany) using the proper size of tongue blade in order to expose the tongue base. Then, the lingual tonsillectomy, including partial trimming of the tongue base musculature, was performed under a 30-degree 3D camera endoscope by the monopolar electrode. The resection area was 1.5 cm posterior to the foramen cecum. The width of resection was 3 cm (1.5 cm apart from mid-line tongue base bilaterally), and the depth of resection was 1.5 cm from the surface of the tongue base. The resection was performed until the epiglottis was visible, without injury to the epiglottis mucosa.

### 2.5. Assessments

Following the normal medical care process, all OSA patients were assessed using polysomnography (PSG) and ESS by the same otolaryngologist before and after the operations. The assessments were conducted in a sleep medicine center of CMUH.

#### 2.5.1. Polysomnography

The standard PSG was used to analyze the patients in accordance with the American Academy of Sleep Medicine (AASM) guidelines [24]. All OSA patients were assessed using PSG and ESS by the same otolaryngologist before and at least six months after surgery. The AHI, minimum SpO_2_%, cumulative time spent below 90% (CT90), and arousal index (AI) were analyzed. AHI was calculated using the sum of apneas and hypopneas by sleep hours and classified as mild (AHI = 5–15), moderate (AHI = 16–29), and severe (AHI ≥ 30) [25]. Surgical success has been traditionally defined as a reduction in the AHI by 50% and AHI < 20 after surgery. The criteria for a treatment cure are defined as an AHI < 5 after treatment.

#### 2.5.2. Epworth Sleepiness Scale

The ESS is a self-administered questionnaire with eight items. A 4-point scale was used to measure the falling asleep probability. The total score of ESS was a range from 0 to 24, and a higher ESS represented the higher daytime sleepiness [26].

#### 2.5.3. Follow-Up Assessments

In the follow-up of clinical care, the patients in both BRP plus TORSTBR and UPPP plus TORSTBR groups were monitored for adverse events, such as post-surgical pain, complications, and removal suture after the surgeries. At the 3-day and 14-day postoperative visits, the post-surgical pain was measured by the Visual Analogue Scale (VAS), scoring from 0 (no pain) to 10 (severe pain) [27]. The records of removal suture 1 month after surgery were collected. The complications of bleeding, dysgeusia, dysphagia, and globus were monitored within one month by one physician.

### 2.6. Statistical Analysis

Statistical analyses were performed using SPSS 25 software (SPSS Inc., Chicago, IL, USA). Data were expressed as mean ± standard deviation. The categorical variables were analyzed by the chi-square test, and continuous variables were compared using the *t*-test. For comparisons of variables before and after interventions, the paired *t*-test was used for analysis. Effect size (d) was calculated in both groups and was classified according to the study of Cohen et al. into very small (d < 0.2), small (0.2 ≤ d < 0.5), medium (0.5 ≤ d < 0.8), and large (d ≥ 0.8) [28]. A *p* < 0.05 was considered statistically significant.

## 3. Results

Among the 62 patients in this analysis, 31 patients received the surgery of BRP plus TORSTBR and 31 patients received the surgery of UPPP plus TORSTBR. There were no significant differences in demographic data between the two groups before the surgery (all *p* > 0.05, Table 1).

In Table 2, after undergoing the operation of BRP plus TORSTBR, the BRP plus TORSTBR group had significantly improved the outcomes of ESS, AHI, minimum SpO_2_%, CT90, and AI (all *p* < 0.05, effect size d = 0.68–1.12). Similarly, in the UPPP plus TORSTBR group, significant improvements in all variables were found after the operation (all *p* < 0.05, effect size d = 0.52–0.97).

Before the operations, there were no significant differences in the patients with different levels of AHI (*p* > 0.05, Table 3). The numbers of patients with normal and abnormal AHI also did not show a significant difference (*p* > 0.05). Compared to the UPPP plus TORSTBR group, the higher increases in AHI reduction, AHI reduction rate, and surgical success were noted in the BRP plus TORSTBR group (all *p* < 0.05). However, there was no significant difference in cure after the operation between the two groups.

For postoperative visits, there were no significant differences in pain VAS between the BRP plus TORSTBR and UPPP plus TORSTBR groups at the 3-day (5.31 ± 3.76 versus 5.74 ± 4.21, *p* = 0.67) and 14-day marks (3.78 ± 2.87 versus 4.32 ± 3.56, *p* = 0.51). The length of hospital stay was not significantly different between the two groups. Within one month, one patient had bleeding (3.22%), one patient had dysgeusia (3.22%), five patients (16.12%) had dysphagia, and seven patients had globus (22.58%) in the BRP plus TORS group. In the UPPP plus TORSTBR group, two patients had bleeding (6.45%), one patient had dysgeusia (3.22%), six patients (19.35%) had dysphagia, and three patients had globus (9.67%). However, there were no significant differences in symptoms of bleeding, dysgeusia, and globus between the two groups (all *p* > 0.05). The records of removal suture after one month were found that eleven patients (35.48%) in BRP plus TORSTBR were needed, and three patients (9.67%) in UPPP plus TORSTBR were needed. No significant difference in records of removal sutures between the two groups was noted (*p* = 0.07).

## 4. Discussion

The incidences of OSA syndrome have increased threefold in the last 20 years [29]. Single-level surgery had a limited number of successful surgical results in the last two decades [29]. UPPP with or without tonsillectomy could not only improve the respiratory events during night sleep but also improve sleep quality, depression, sexual function, ventricular function, and promote safe driving in OSA patients [30]. However, the surgery for moderate and severe OSA by UPPP base therapy produced a limited successful outcome. The BMI, AHI severity, age of patient, pattern of airway collapse, experience of the surgeon, and even the patient’s choice all affect the treatment outcome [31]. In addition, the tongue base is addressed more by sleep physicians in moderate and severe OSA patients in the practice of drug-induced sleep endoscopy. Therefore, managing the tongue base could result in further treatment for surgical failure by palatal surgery only. Vicini et al. applied the robotic tongue base surgery combined with ESP to treat OSA patients and reported a higher surgical success rate [15]. Besides, various palatal surgeries that are combined with TORS produce a higher surgical success rate, as found by Cammaroto et al. [16]. 

Managing the tongue is not only a diagnostic issue, it also affects the surgical treatment strategy. The unresolved sleep apnea forces the sleep physicians to apply the drug-induced sleep endoscopy to find out the anatomic obstruction site of the upper airway to surgically solve the OSA. In the current study, we compared the effects on ESS, AHI, minimum SpO_2_%, CT90, and AI in BRP plus TORSTBR and UPPP plus TORSTBR treatment groups. Significant improvements after BRP or UPPP plus TORSTBR were found in the patients with moderate to severe OSA, and medium to large effect sizes in the BRP plus TORSTBR group (d = 0.54–0.81) and UPPP plus TORSTBR group (d = 0.52–0.97) were revealed. In the recent literature, simultaneous retropalatal and retroglossal collapse or obstruction are frequently found in 25–33% of OSA cases [32,33,34]. Therefore, multilevel surgery for patients with OSA should be considered to achieve more effective outcomes than single-level surgery. Multilevel surgery rendered a 66% surgical success rate offered by Lin et al. [35]. Our results revealed that the surgery of BRP plus TORSTBR had a better AHI reduction rate and surgical success rate on moderate to severe OSA than the operation of UPPP plus TORSTBR, although outcomes were not significantly different since both methods reduced disease severity in ESS, AHI, minimum SpO_2_%, CT90, and AI, measured by post-operation PSG. Concerning palatal surgery as a part of the multilevel surgery, there was still residual obstruction at the retropalatal level, even after palatal surgery. Thus, plenty of crucial innovations for palatal surgery are offered as relocation pharyngoplasty, expansion sphincter palatoplasty, and suspension palatoplasty in order to achieve higher surgical success [36,37]. The number of palatal surgeries performed with barbed suture has been rising recently due to the innovation in suture stitches by v-loc sutures. The barbed suture in pharyngoplasty was demonstrated by Mantovani et al. in 2013 [38] and Salamanca et al. in 2014 [39], and following this, pharyngoplasty performed with barbed suture increased and became more widely recommended. Barbed reposition pharyngoplasty in multilevel surgery was noted by Vicini et al., as it could conduct a widening of the oropharyngeal lateral wall and forward sustaining of the soft palate; it was also faster, easier, and more feasible within the robotic surgery framework [40].

Barbed reposition pharyngoplasty has had the same effect as expansion sphincter pharyngoplasty combined with anterior palatoplasty in terms of enlarging both the lateral and anteroposterior direction of retropalatal space in the study by Babademez et al. [41]. Barbed suspension pharyngoplasty was shown in a 2019 study by Barbieri et al., and the study compared barbed reposition pharyngoplasty and barbed suspension pharyngoplasty [42]. Both surgeries had the same excellent result. The BRP was the less invasive procedure for preserving palatopharyngeal muscle than barbed reposition pharyngoplasty, and the comparisons of cured rates between BRP and BRP were not statically significant [42]. In our study, we selected patients with moderate to severe OSA and proved multilevel collapse (retropalatal and retroglossal spaces collapse) for multilevel surgery by DISE. After enrolling these patients, we completed multilevel surgery, including the palatal surgery with TORSTBR to compare its effects in different palatal surgeries (BRP versus modified UPPP). We analyzed the pre-operative and postoperative polysomnography data, Epworth sleepiness scale, tonsil grade, and the Friedman tongue position between the BRP and UPPP groups.

**Figure 3:**
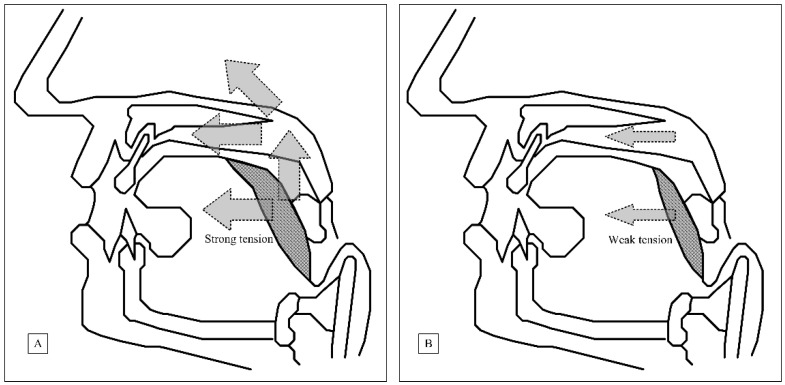
The palatal suture mechanism of BRP plus TORSTBR (**A**) and UPPP plus TORSTBR (**B**) techniques.

Figure 3 illustrates the difference in palatal suture mechanism of BRP plus TORSTBR and UPPP plus TORSTBR techniques. The barbed palatal suture offered a good quality of posterior tonsillar pillar suspension and lateralization vector to increase not only anterior–posterior diameter but also to widen the lateral space of the retropalatal area [43]. In addition, the palatoglossus muscle suture being fixed to the pterygo-mandible-raphy offered the opportunity for the suspension vector to keep the tongue base from dropping. Therefore, we were able to not only widen the retropalatal space but also increase the anterior–posterior diameter of the retroglossal area [44]. Our findings were similar to the results of Cammaroto et al., and the effect of the barbed palatal suture mechanism was proven [16].

The pre-operative data between the two groups were not significantly different. The postoperative AHI and ESS were significantly decreased in both groups, and multilevel surgery was considered to be effective. The surgical success rate of the BRP plus TORSTBR group (67.74%) was significantly superior to UPPP plus TORSTBR group (38.71%), and it indicated that the different palatal surgery performed in multilevel OSA surgery had a different effect on surgical success. BRP is superior to modified UPPP in multilevel surgery for moderate to severe OSA patients. The results of our study are similar to the outcomes of Cammaroto et al. [16]. Modified UPPP with TORSTBR had a poor success rate of 38.71%. In our patient data, the Friedman tongue position in the UPPP group is not significantly severe compared to those in the BRP group. However, a higher surgical success rate is obtained in the BRP group. Therefore, we consider BRP to be more suitable as a part of multilevel surgery for moderate and severe OSA in managing the retroglossal space narrowing related OSA.

Barbed surgery had an advantage in terms of reduced operative time (less knot time), less knot rupture, more stiffness of the soft palate, and fewer minor complications, such as extruded thread, bleeding, suture rupture, and pharyngoplasty dehiscence [45]. Barbed suspension pharyngoplasty in multilevel surgery is the more feasible, faster, and less invasive method [46]. However, further study is warranted for comparing BRP to lateral pharyngoplasty and extension sphincter pharyngoplasty to treat moderate and severe OSA. In the current study, the complications included post-operational pain, bleeding, dysgeusia, transient dysphagia, throat globus sensation, and the need to remove prolonged stitches after one month; all showed no significant differences between the BRP plus TORSTBR versus UPPP plus TORSTBR groups (*p* > 0.05). Although there was a higher rate of prolonged stitches that needed to be removed one month after surgery, no significance could be found. Thus, we need to care for the prolonged stitches in patients who receive barbed suspension palatoplasty.

There are some limitations to the current study. The small sample size is one of the main limits of our study, and the non-parametric approach means that we were unable to adjust for potential confounders. Long-term results of barbed suspension pharyngoplasty in multilevel surgery are warranted. In the future, prospective, randomized, and controlled trials that incorporate similar surgical techniques will be needed to evaluate the efficacy of different palatal surgeries in multilevel surgery.

## 5. Conclusions

In our study, BRP with TORSTBR was a feasible, faster, and effective multilevel surgery for moderate to severe OSA. Modified UPPP might be less effective compared to BRP as a part of multilevel surgery for moderate to severe OSA.

## Figures and Tables

**Table 1:** Demographic data of the two groups.

	BRP Plus TORSTBR Group (*n* = 31)	UPPP Plus TORSTBR Group (*n* = 31)	*p*
Age	37.51 ± 9.42	39.61 ± 11.63	0.59
Male/female	26/5	24/7	0.52
Body mass index	28.22 ± 3.19	28.20 ± 3.62	0.88
Preop ESS	9.02 ± 4.57	11.02 ± 4.58	0.28
Tonsil grade	1.93 ± 1.14	2.11 ± 1.38	0.55
FTP	2.92 ± 0.66	3.01 ± 0.55	0.22
Preop AHI	46.35 ± 21.76	48.24 ± 21.18	0.69

UPPP, uvulopalatoplasty; BRP, barbed repositioning pharyngoplasty; TORSTBR, transoral robotic surgery tongue base reduction; FTP, Friedman Tongue Position; AHI, apnea–hypopnea index.

**Table 2:** Within-group comparison of the treatment outcomes.

	BRP Plus TORSTBR Group (*n* = 31)				UPPP Plus TORSTBR Group (*n* = 31)			
	Pre-Op	Post-Op	*p*	Effect Size	Pre-Op	Post-Op	*p*	Effect Size
ESS	9.03 ± 4.52	6.60 ± 3.82	0.02 *	0.58	11.01 ± 4.52	7.82 ± 3.45	0.01 *	0.79
AHI	46.21 ± 22.03	21.60 ± 21.54	0.001 *	1.12	45.13 ± 19.31	28.75 ± 23.09	0.04 *	0.76
Minimum SpO_2_%	76.44 ± 7.63	80.51 ± 7.33	0.02 *	0.54	75.12 ± 7.66	82.56 ± 7.64	0.02 *	0.97
CT90	16.32 ± 17.13	6.95 ± 10.46	0.001 *	0.66	14.24 ± 14.65	7.54 ± 10.37	0.03 *	0.52
AI	31.66 ± 23.53	14.39 ± 18.34	0.001 *	0.81	33.3 ± 19.24	16.5 ± 17.57	0.01 *	0.91

* *p* < 0.05. CT90, cumulative time spent below 90%; AI, arousal index; UPPP, uvulopalatoplasty; BRP, barbed repositioning pharyngoplasty; TORSTBR, transoral robotic surgery tongue base reduction; AHI, apnea–hypopnea index.

**Table 3:** Between-group comparison of the treatment outcomes.

	BRP Plus TORSTBR Group (*n* = 31)	UPPP Plus TORSTBR Group (*n* = 31)	*p*
Pre-op AHI			
Mild (AHI 5–15) (*n*, %)	0 (0%)	0(0%)	0.75
Moderate (AHI 16–30)(*n*, %)	11 (35.48%)	10 (32.25%)	
Severe (AHI > 30)(*n*, %)	20 (64.51%)	21 (67.74%)	
Postop AHI			
Normal (AHI < 5)(*n*, %)	6 (19.35%)	8 (25.80%)	0.54
Abnormal(AHI ≥ 5) (*n*, %)	25 (80.64%)	23 (74.19%)	
AHI reduction	24.73 ± 10.46	17.34 ± 14.82	0.04 *
AHI reduction rate (%)	62.01 ± 3.03	43.07 ± 9.06	0.01 *
Outcome			
Cure (*n*, %)	6 (19.35%)	5 (16.12%)	0.69
Surgical success (*n*, %)	21 (67.74%)	12 (38.71%)	0.02 *
Comorbidities			
Bleeding (*n*, %)	1 (3.22%)	2 (6.45%)	0.55
Dysgeusia (*n*, %)	1 (3.22%)	1 (3.22%)	1.00
Dysphagia (*n*, %)	5 (16.12%)	6 (19.35%)	0.73
Globus (*n*, %)	7 (22.58%)	3 (9.67%)	0.16

* *p* < 0.05. UPPP, uvulopalatoplasty; BRP, barbed repositioning pharyngoplasty; TORSTBR, transoral robotic surgery tongue base reduction; AHI.

## Data Availability

Data is contained within the article.

## Abbreviations

OSA	obstructive sleep apnea
TDI	threshold, discrimination, and identification
FTP	Friedman Tongue Position
CT90	cumulative time spent below 90%
AI	arousal index
DISE	drug-induced sleep endoscopy
UPPP	uvulopalatoplasty
BRP	barbed repositioning pharyngoplasty
TORS	transoral robotic surgery
TORSTBR	transoral robotic surgery tongue base reduction
AHI	apnea–hypopnea index
PSG	polysomnography
AASM	American Academy of Sleep Medicine
VAS	Visual Analogue Scale
